# Intracerebral Hemorrhage During the Chronic Phase of Eosinophilic Granulomatosis With Polyangiitis: A Case Report Emphasizing Cerebrovascular Vulnerability

**DOI:** 10.1002/ccr3.71944

**Published:** 2026-01-24

**Authors:** Shinya Watanabe, Masatoshi Kasuya, Yasushi Shibata

**Affiliations:** ^1^ Department of Neurosurgery Mito Kyodo General Hospital, Tsukuba University Hospital Mito Area Medical Education Center Mito Ibaraki Japan

**Keywords:** anticoagulation therapy, central nervous system involvement, disease remission, eosinophilic granulomatosis with polyangiitis, intracerebral hemorrhage, putaminal hemorrhage

## Abstract

Eosinophilic granulomatosis with polyangiitis (EGPA) is a rare form of small‐vessel vasculitis typically associated with eosinophilia, asthma, and systemic inflammation. Although peripheral neuropathies are relatively common, central nervous system involvement, especially intracerebral hemorrhage during the remission phase of EGPA, is uncommon, and its clinical course and management remain poorly understood. The female patient had been diagnosed with EGPA 6 months earlier based on marked eosinophilia of 16,380/μL, late‐onset asthma, and mononeuritis multiplex, fulfilling the 2022 American College of Rheumatology/European Alliance of Associations for Rheumatology classification criteria. After receiving high‐dose corticosteroids (Day‐165) and edoxaban for subacute distal deep vein thrombosis (Day‐158), her eosinophil count steadily decreased, and she was discharged without hypertension on oral prednisolone 40 mg/day (Day‐144). The dose was tapered to 13 mg/day without relapse. On Day 0, she developed acute left putaminal hemorrhage along with previous cerebral microbleeds on susceptibility‐weighted imaging (SWI) and an old, small brainstem infarction. Laboratory data showed a normal eosinophil count of 70/μL and normal D‐dimer levels. Edoxaban was discontinued, and conservative management was initiated. Follow‐up ultrasound on Day 19 confirmed an organized thrombus without progression. She gradually improved with rehabilitation and achieved a modified Rankin Scale score of 1. Although mild sensory disturbance in the right upper and lower extremities persisted, she regained full independence in all activities of daily living and is currently working energetically as a bookstore clerk. She has remained stable for over 3.5 years without anticoagulation or EGPA relapse. This case highlights that, although perhaps overlooked during the remission phase when clinicians may be less careful, intracerebral hemorrhage may occur even during EGPA remission, potentially owing to persistent small‐vessel endothelial vulnerability, which may elevate hemorrhagic risk even during clinical remission. The presence of microbleeds and prior infarction may reflect longstanding vascular injury, possibly exacerbated by anticoagulation. Current guidelines do not address this risk, underscoring the need for individualized risk assessment. This case may indicate that brain imaging, including SWI, could be considered prior to initiating anticoagulation therapy in patients with EGPA.

## Key Clinical Message

Intracerebral hemorrhage may occur even during the remission phase of eosinophilic granulomatosis with polyangiitis. Preanticoagulation neuroimaging, particularly susceptibility‐weighted imaging, may aid in evaluating cerebrovascular fragility and guiding individualized risk–benefit decisions.

## 1 Introduction

Eosinophilic granulomatosis with polyangiitis (EGPA), formerly Churg‐Strauss syndrome, is a systemic necrotizing vasculitis predominantly affecting small‐ to medium‐sized vessels [1]. The disease is characterized by eosinophilia, asthma, and vasculitic manifestations [1, 2]. The annual incidence ranges from 0.5 to 4.2 per million, with central nervous system (CNS) involvement reported in only 5% of cases [1, 3]. While cerebral infarctions are more common, intracerebral hemorrhage occurs infrequently and usually during periods of active disease [1, 4]. Recent guidelines [1] mention intracerebral hemorrhage during the acute phase but not the chronic phase. Here, we report a rare case of intracerebral hemorrhage occurring during the chronic remission phase of EGPA, underscoring the importance of cerebrovascular risk assessment even when systemic disease activity appears controlled.

## 2 Case History/Examination

The patient, a nonsmoker with no history of lifestyle‐related diseases such as diabetes mellitus or dyslipidemia, had been diagnosed with EGPA at age 51 according to the 2022 American College of Rheumatology/European Alliance of Associations for Rheumatology (ACR/EULAR) classification criteria [5]. She met the criteria with marked eosinophilia of 16,380/μL (normal range, 70–440/μL), asthma, sinusitis, and multiple peripheral neuropathies. Initial treatment began with intravenous steroid pulse therapy (Day‐165). During screening, subacute peripheral deep vein thrombosis (DVT) in the left soleus muscle was identified, and edoxaban 30 mg/day was initiated (Day‐158). After confirming a steady decrease in eosinophil count, the corticosteroid dose was tapered to 40 mg/day, and she was discharged home with a modified Rankin Scale (mRS) score of 1 (Day‐144). Throughout admission, no hypertension was observed, and she was not on any antihypertensive medication. The prednisolone dose was gradually reduced to 13 mg/day without recurrence of eosinophilia.

## 3 Differential Diagnosis, Investigations and Treatment

**FIGURE 1:**
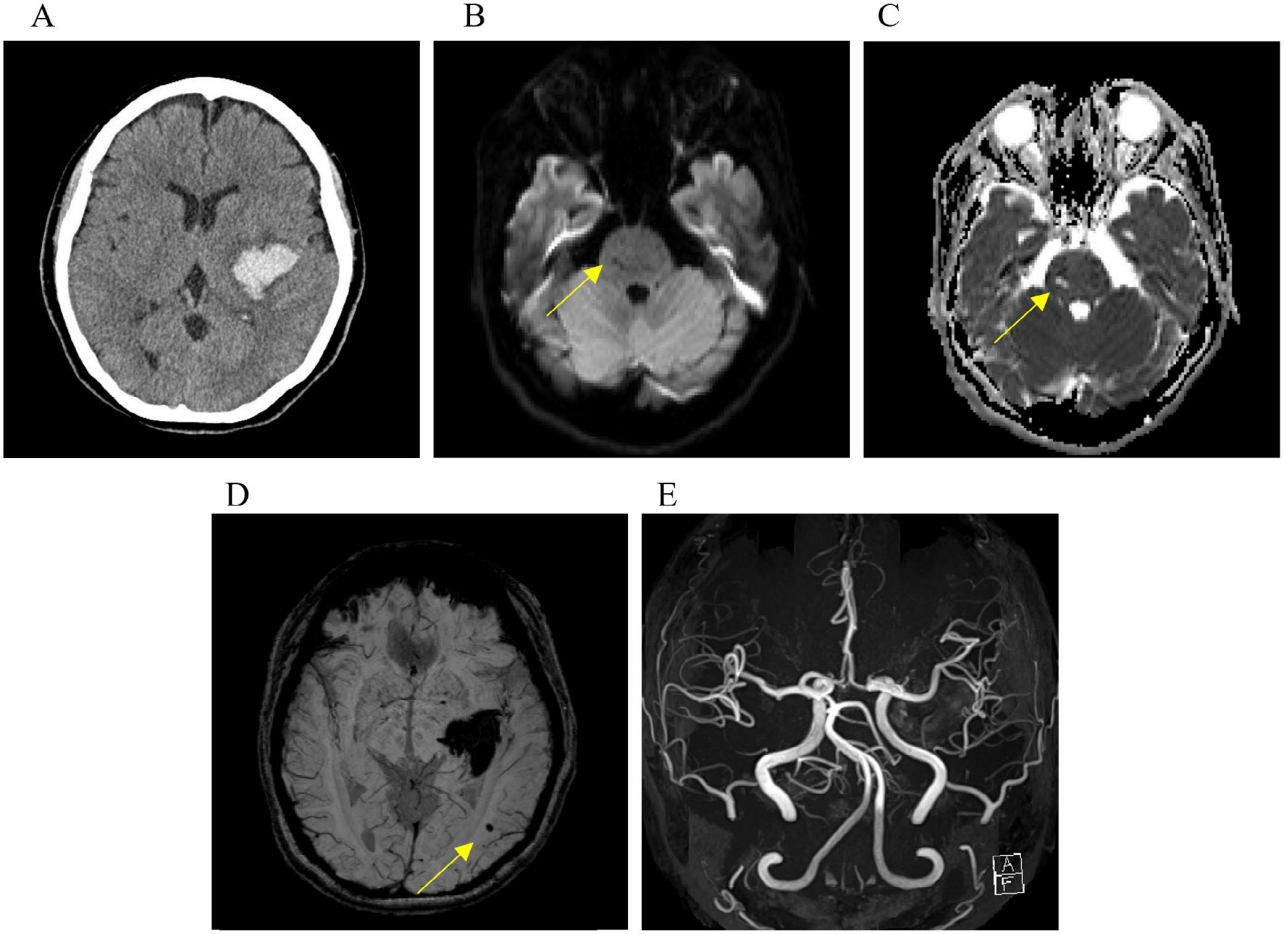
Computed tomography (A) showing high‐signal lesions in the left putaminal region. Diffusion‐weighted (B) and apparent diffusion coefficient magnetic resonance images (C) revealing an old small brainstem infarction. Susceptibility‐weighted imaging (D) showing microbleeds. No stenosis or irregularities of large cerebral arteries were observed on magnetic resonance angiography (E).

At age 52, she presented with sudden‐onset headache, dysarthria, and right hemiparesis (Day 0). Upon emergency admission, she was alert with a Glasgow Coma Scale score of 15. Neurological examination revealed mild right facial palsy, dysarthria, and right‐sided hemiparesis (manual muscle test: 1/5 proximal, 3/5 distal). Sensory deficits were present on the right side of the body. Laboratory tests showed no eosinophilia, with eosinophils at 70/μL (normal range, 70–440/μL), and a D‐dimer of 0.4 μg/mL (normal range, ≤ 1.0 μg/mL), suggesting no active thrombosis or coagulopathy. Computed tomography revealed a left putaminal hyperdensity consistent with putaminal hemorrhage (Figure 1A). Magnetic resonance imaging (MRI), including diffusion‐weighted and apparent diffusion coefficient (ADC) images (Figure 1B,C), showed an old small brainstem infarction. Susceptibility‐weighted imaging (SWI) (Figure 1D) demonstrated a microbleed in the left occipital lobe, suggesting chronic small‐vessel vasculopathy likely due to prior eosinophilic injury. Magnetic resonance angiography (MRA) (Figure 1E) revealed no large‐vessel abnormalities (Day 4).

Ultrasound of the lower extremity confirmed that the previously identified left distal deep vein thrombosis had become organized, with no evidence of new thrombus formation or progression (Day 19). Given the chronic, distal, organized thrombosis, the absence of ongoing thrombosis, normal D‐dimer levels, and the potential risk of further hemorrhage in a patient with suspected microvascular vulnerability, we elected to discontinue edoxaban. This decision was made after weighing the low thrombotic burden against the increased hemorrhagic risk associated with continued anticoagulation. She underwent conservative management, including early rehabilitation. Her neurological deficits gradually improved, and she was transferred for rehabilitation with a mRS score of 3 (Day 36).

## 4 Conclusion and Results (Outcome and Follow‐Up)

She has remained free of recurrent intracerebral hemorrhage and EGPA flares for 3.5 years under maintenance therapy with low‐dose prednisolone. Notably, no recurrence of DVT has been observed during this period without anticoagulation. She currently has an mRS of 1, has returned to work as a bookstore clerk, is independent in all activities of daily living, and reports only mild residual right‐sided clumsiness without motor weakness.

The patient was initially distressed by the onset of hemiparesis due to cerebral hemorrhage, especially as her EGPA had been well controlled. However, she has gradually come to terms with her condition. Written informed consent was obtained from the patient for both participation and publication, including the use of accompanying images.

## 5 Discussion

### 5.1 EGPA Classification and Central Nervous System Involvement

EGPA is a small‐ to medium‐vessel necrotizing vasculitis characterized by eosinophilia, asthma, and systemic manifestations [1]. According to the 2022 ACR/EULAR classification criteria for EGPA [5], a total score of 6 or more supports classification in patients with suspected vasculitis. In the present case, the patient fulfilled these criteria with late‐onset asthma (+3), marked peripheral eosinophilia (+2), and mononeuritis multiplex (+1), totaling 6 points. She was negative for cytoplasmic anti‐neutrophil cytoplasmic antibodies (ANCA) and had no hematuria (0 points each). Although these criteria are primarily intended for classification in research settings, the patient's clinical and laboratory features were highly consistent with EGPA, supporting the working diagnosis even in the absence of histopathological confirmation. While histological confirmation was not available, the diagnosis was strongly supported by the 2022 ACR/EULAR criteria. Nevertheless, we acknowledge that classification criteria are not diagnostic tools per se, and alternative eosinophilic disorders should always be considered.

CNS involvement in EGPA is relatively uncommon, occurring in approximately 5%–9% of EGPA cases [3, 4], and typically manifests during the active inflammatory phase, which is usually associated with active vasculitic lesions, hypertension, or eosinophilic infiltration [1, 5, 6]. Among cases of CNS involvement, cerebral infarction accounted for 50%, while intracerebral hemorrhage occurred in 10% [4]. Most reports of cerebral hemorrhage occurred during active disease. In contrast, hemorrhagic events during clinical remission are rare and sparsely documented. Thus, our case may have meaningful implications for future management strategies during the remission phase of EGPA.

### 5.2 Persistent Vascular Risk and Anticoagulation in Remission‐Phase EGPA

This case highlights a spontaneous intracerebral hemorrhage occurring during the chronic phase of EGPA, with normalized eosinophil counts and stable blood pressure. This unexpected event may indicate that structural vascular vulnerability, possibly due to prior eosinophil‐mediated endothelial injury, may persist even during remission. In this context, “vulnerability” encompasses both residual microvascular damage and impaired vascular integrity, which may not be apparent on conventional imaging but can predispose patients to hemorrhagic events under anticoagulation. One plausible mechanism is subclinical endothelial damage from earlier eosinophil activity. Eosinophils release cytotoxic granule proteins such as major basic protein and eosinophil cationic protein, which can damage vascular endothelium, increase permeability, and promote thrombogenicity even in the absence of active inflammation [7]. In our case, SWI revealed cerebral microbleeds, and ADC imaging showed a chronic lacunar infarction in the brainstem, further supporting the presence of small‐vessel disease and latent vascular vulnerability.

Another important contributing factor may be anticoagulation. Although edoxaban was appropriately initiated for distal DVT, its continuation may have unmasked preexisting subclinical vasculopathy, increasing susceptibility to hemorrhage. While a direct causal relationship cannot be confirmed, anticoagulation may have acted as a precipitating factor. Notably, a large multicenter cohort study failed to demonstrate a statistically significant reduction in thromboembolic risk with anticoagulant therapy in EGPA patients (hazard ratio: 0.35, 95% confidence interval: 0.09–1.57), highlighting uncertainty regarding its benefit in this population [8]. A prior case report also described multiple cerebral infarctions and concurrent subarachnoid hemorrhage in a similar context [9]. These findings may indicate that conventional anticoagulation risk stratification may not adequately address residual vascular inflammation or structural vulnerability, even in ANCA‐negative and eosinophil‐normal states [3, 5]. From a diagnostic standpoint, other common causes of intracerebral hemorrhage—including vascular malformations, coagulopathy, and hypertensive crisis—were not supported by imaging and laboratory evaluations. The localization of hemorrhage to the putamen, a region often affected by small‐vessel disease, may further reflect underlying vasculitic microangiopathy [6]. Although the hemorrhage was located in the putamen, a typical site of hypertensive hemorrhage, the absence of a history of hypertension and the lack of microvascular changes on prior MRI approximately 4 years earlier argue against hypertensive hemorrhage as the primary cause in this case.

### 5.3 Clinical Implications and the Need for Cerebrovascular Vulnerability

Importantly, this case reinforces the growing recognition that clinical remission in EGPA does not necessarily equate to restored vascular integrity. Long‐term endothelial sequelae may persist and influence future cerebrovascular risk [7]. Therefore, caution is warranted when initiating or continuing anticoagulation in EGPA patients, even during clinically quiescent phases. Prospective studies are required to determine the incidence and risk factors of CNS hemorrhage in vasculitis patients receiving anticoagulation. Notably, current evidence‐based guidelines do not address this risk. While recent guidelines [1] recognize CNS involvement as a severe manifestation requiring aggressive induction therapy, they do not discuss the risk of delayed hemorrhage during remission or under anticoagulation. In this case, prior to EGPA diagnosis, a brain MRI was performed as part of a workup for dizziness (Day‐1230), revealing only mild white matter changes without evidence of microvascular lesions such as brainstem infarctions. However, MRI was not performed at the time of EGPA diagnosis. This case underscores the importance of continued cerebrovascular vulnerability in vasculitis patients, even during clinical remission and while on anticoagulant therapy. In light of these findings, brain MRI, including SWI and MRA, may be considered prior to initiating anticoagulation in EGPA patients, particularly those with prior neurological symptoms or known vasculitic involvement. Early detection of microvascular pathology may help guide individualized risk–benefit decisions.

This case report was prepared in accordance with the CARE (CAse REport) guidelines [10], ensuring comprehensive and transparent reporting of clinical information.

## 6 Conclusion

This case illustrates that intracerebral hemorrhage can occur in EGPA patients even during remission, particularly in the context of anticoagulation. The findings underscore the importance of individualized risk assessment prior to initiating anticoagulant therapy, even during clinically quiescent phases. This case further highlights the need for ongoing cerebrovascular monitoring in EGPA patients. Accordingly, preanticoagulation brain MRI, including SWI, may be a valuable tool to assess baseline cerebrovascular vulnerability in EGPA patients.

## Author Contributions

**Shinya Watanabe:** data curation, formal analysis, investigation, resources, validation, visualization, writing – original draft, writing – review and editing. **Masatoshi Kasuya:** conceptualization, formal analysis, resources, software, visualization, writing – original draft, writing – review and editing. **Yasushi Shibata:** conceptualization, methodology, project administration, resources, supervision, writing – review and editing.

## Funding

The authors have nothing to report.

## Disclosure

Permission to reproduce material from other sources: This manuscript does not include any material that requires permission for reproduction from other sources.

## Ethics Statement

The authors have nothing to report.

## Consent

Written informed consent was obtained.

## Conflicts of Interest

The authors declare no conflicts of interest.

## Data Availability

The data supporting the findings of this case are available upon request.
